# Navigating Through ARCP and CT Portfolio

**DOI:** 10.1192/bjo.2025.10275

**Published:** 2025-06-20

**Authors:** Anjali Ghosalkar

**Affiliations:** Surrey and Borders Partnership NHS Foundation Trust, Surrey, United Kingdom

## Abstract

**Aims:** To support psychiatry core trainees in navigating their core training, ensuring a smooth and successful ARCP outcome, and facilitating the development of a comprehensive CT portfolio for competitive HST applications.

**Methods:** We analysed data from the Royal College of Psychiatrists’ Gold and Silver Guides, supplemented by additional relevant resources. Based on this, we developed scripts and storyboards for an educational short film, currently in progress, and designed an accompanying educational poster to present the key information.

**Results:** The analysis led to the identification of key training strategies and resources critical for psychiatry core trainees. The development of the educational short film and poster has facilitated clear, accessible guidance, enhancing trainee understanding of the ARCP process and HST application requirements.

**Conclusion:** The educational resources developed offer valuable support to psychiatry core trainees, aiding in ARCP preparation and HST applications. Continued refinement of these tools will further enhance their utility.

